# Theoretical Modeling of Long-Time Drug Release from Nitrosalicyl-Imine-Chitosan Hydrogels through Multifractal Logistic Type Laws

**DOI:** 10.1155/2019/4091464

**Published:** 2019-08-14

**Authors:** Anda-Mihaela Craciun, Mihaela Luminita Barhalescu, Maricel Agop, Lăcrămioara Ochiuz

**Affiliations:** ^1^Petru Poni Institute of Macromolecular Chemistry, GrigoreGhicaVoda Alley, Iasi, Romania; ^2^Constanta Maritime University, Department of General Engineering Sciences, 104 Mircea cel Batran Street, 900663 Constanta, Romania; ^3^Gheorghe Asachi Technical University of Iasi, Faculty of Machine Manufacturing and Industrial Management, Department of Physics, D. Mangeron Bvd. No. 73, 700050 Iasi, Romania; ^4^Romanian Scientists Academy, 54 Splaiul Independentei Blvd., Bucharest 050094, Romania; ^5^Faculty of Pharmacy, Department of Pharmaceutical Technology University of Medicine and Pharmacy “Gr.T. Popa”, Iasi, Romania

## Abstract

Drug release is a complex phenomenon due to the large number of interdependent side effects that occur simultaneously, involving strong nonlinear dynamics. Therefore, since their theoretical description is difficult in the classical mathematics modelling, we have built a theoretical model based on logistic type laws, validated by the correlations with the experimental data, in a special case of drug release from hydrogels. The novelty of our approach is the implementation of multifractality in logistic type laws, situation in which any chaotic system, characterized by a small number of nonlinear interactions, gets memory and, implicitly, characterization through a large number of nonlinear interactions. In other words, the complex system polymer-drug matrix becomes “pseudo-intelligent.”

## 1. Introduction

Hydrogels are an important class of materials with a large range of applications in key domains as biomedicine [1], hygiene [2], environment protection [3], agriculture [4], and so on. Among the hydrogels, those based on natural and biopolymers are of crucial significance for bioapplications due to their biocompatibility and biodegradability, mandatory features for their safe in vivo use and for limiting the environmental pollution [5–7]. In this regard, the most suited polymer for preparation of the hydrogels targeting bioapplication is chitosan, a biopolymer derived from the natural polymer chitin [8]. The chitosan endows the hydrogels containing it, along with the required biocompatibility and biodegradability, with other remarkable properties as antitumor, antimicrobial, antifungal, fat binding and, very important, with the ability to encapsulate a large range of drugs by strong physical forces for a further prolonged delivery. Moreover, due to its intrinsic properties, the use of chitosan-based hydrogels as drug matrix led to multifunctional systems, with enhanced biological possibilities, which could be further improved by a proper choice of the chitosan crosslinker [9–15].

Another important aspect related to the developing of drug delivery systems is related to the efficient use of the drugs, limiting thus their side effects on (i) the human body and (ii) the environment. As an example, many antitumor drugs, e.g., 5-fluorouracil, which are extensively used in the treatment of a wide variety of tumor lines [9], showed that their systemic administration led to the interference with the structure and function of DNA of the tumor cells but unfortunately affects also nontarget normal cells. Moreover, part of them is excreted in the environment, and recent studies revealed their toxicity and genotoxicity into zebrafish, indicating them as potential negative contaminants [16]. This is the reason why the researchers focus their attention to the local control release of the antitumor drugs, which should limit their side effects on the human body and on the environment also.

In this context, targeting the preparation of hydrogels for the local therapy of cancer, chitosan has been reacted with nitrosalicylaldehyde, when biocompatible hydrogels with high cytotoxicity against the HeLa cells were obtained [11]. Moreover, the hydrogels demonstrated fast hydrogelation in physiological pH and moderate swelling while maintaining their shape, features which recommend them for local therapy of cancer. Taking into consideration these findings, they were further investigated as matrix for drug delivery systems, when demonstrating prolonged drug release [17]. For a better understanding of the drug release mechanism, we proposed to model the drug release kinetics both by means of empiric laws and by means of logistic type laws.

The novelty of our approach is the implementation of multifractality in logistic type laws for the description of the drug release processes. In such a situation, the complex system polymer-drug matrix gets memory, becoming “pseudo-intelligent.”

## 2. Experimental

### 2.1. Materials

Chitosan of low-molecular weight, nitrosalicylaldehyde of 98% purity, diclofenac sodium salt (DCF) of purity higher of 99%, and phosphate buffer solution were purchased from Aldrich and used as received.

### 2.2. Preparation of Drug Delivery Systems

**Figure 1:**
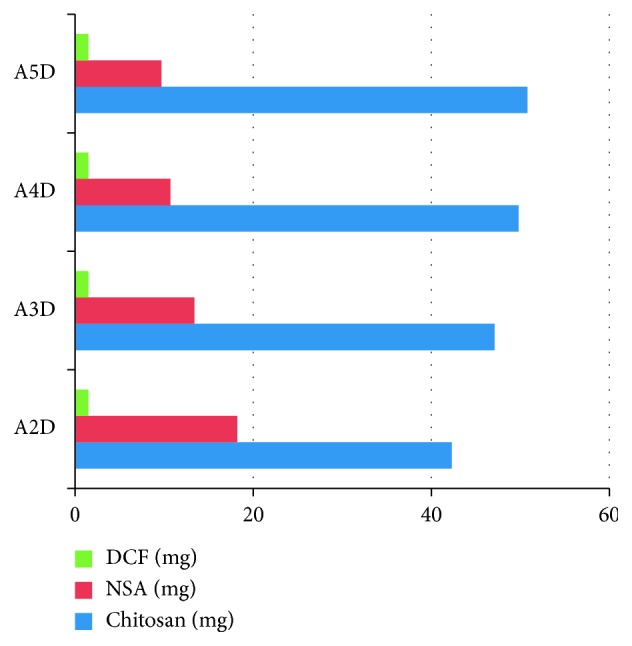
Graphical representation of the reagents weight used for hydrogel preparation.

The drug delivery systems which are the subject of the present paper were obtained according to a procedure already reported [17]. Shortly, they were prepared by in situ hydrogelation of chitosan with nitrosalicylaldehyde (NSA) in the presence of diclofenac sodium salt (DCF), based on the imination reaction along with the self-ordering of the newly formed imine units [11]. In such a way, the new imine units formed by the reaction of amine units of chitosan with nitrosalicylaldehyde segregated into ordered clusters which played the role of crosslinking nodes. Varying the molar ratio of the amine/aldehyde functional units, a series of four drug delivery systems A2D–A5D were prepared (Figure 1). It should be mentioned here that the amount of drug has been kept constant, while the amount of chitosan and nitrosalicylaldehyde was varied in order to obtain drug delivery systems with different crosslinking densities, which should influence the drug release mechanism.

### 2.3. In Vitro Investigation of the Drug Release

**Figure 2:**
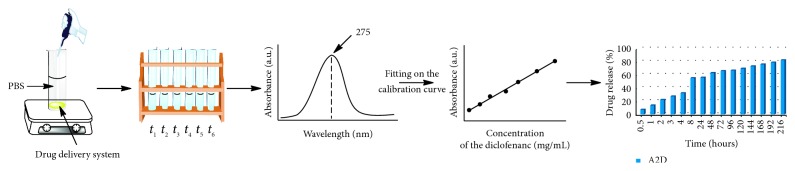
In vitro drug release investigation.

In order to monitor the drug delivery profile of the studied formulations, equal amounts of the synthetized A2D–A5D systems were prepared as pills by pressing with a hydraulic press (2 N/m^2^). The pills were introduced into sealed vials containing 10 ml of PBS at 37°C. At predetermined moments, 2 mL of supernatant was withdrawn and replaced with 2 mL of PBS. The supernatant samples were collected and analyzed by quantitative absorption spectroscopy, by measuring the absorbance of the DCF drug. The values of the absorbance obtained at different investigation times were fitted on the DCF calibration curve [9, 17]. The cumulative release of the 5FU was estimated from the Beer–Lambert law. All the experiments were realized in triplicate. The absorbance spectroscopy was run on a Horiba spectrophotometer. A schematic representation of the in vitro drug release investigation is given in Figure 2.

## 3. Theoretical Considerations

The release kinetics of the studied formulations was first investigated by fitting the resulted release data on the equations of 5 different models: *zero order, first order, Higuchi, Korsmeyer-Peppas,* and *Hixson–Crowell* [18]. Their equations are presented below:

- Zero-order model: *M*_*t*_=*K*_0_*t*, where *M*_*t*_ is the amount of DCF released in the time *t* and *K*_0_ is the zero-order release constant
- First-order model: log  *M*_*t*_=*Kt*/2.303, where *M*_*t*_ is the amount of DCF released in the time *t* and *K*_0_ is the zero-order release constant
- Higuchi model: *M*_*t*_=*K*_*H*_*t*^(1/2)^, where *M*_*t*_ is the amount of DCF released in the time *t* and *K*_*H*_ is the Higuchi dissolution constant
- Korsmeyer–Peppas model: (*M*_*t*_/*M*_*∞*_)=*Kt*^*n*^, where (*M*_*t*_/*M*_*∞*_) is the fraction of DCF released at the time *t*, *K* is the release rate constant, and *n* is the release exponent
- Hixson–Crowell model: *W*_0_^1/3^ − *W*_*t*_^1/3^=*K* · *t*, where *W*_0_ is the initial amount of DCF, *W*_*t*_ the remaining amount of DCF at time *t* and *K* a constant.

Due to the empirical character of these equations, the release kinetics was further investigated through nonlinear dynamics in the form of logistic type laws.

The polymeric matrix loaded with drug is, both structurally and functionally, a complex system, hereafter referred to as polymer-drug matrix. In this case, the complexity refers both to the collective behaviour of the polymer-drug matrix (dictated by the extremely high number of nonlinear interactions between its structural units) and to the constraints that the polymeric-drug matrix system supports in relation to the “environment” generated by various biological structures. From such a perspective, the polymer-drug matrix evolves away from the state of equilibrium (at the “edge of chaos” between determinism and randomness), in a critical state built from an “archeology”/history of “unpredictable and unexpected events” through feedback cycles, self-organized structures, etc., thus, the archeology/history is grounded as the main feature of the polymer-drug matrix.

**Figure 3:**
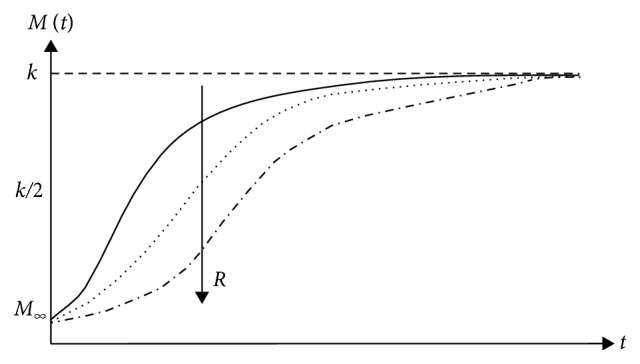
Release curves of the same *k*〉*M*_*∞*_ and various *R* described by the solution of the logistic type equation (1).

**Figure 4:**
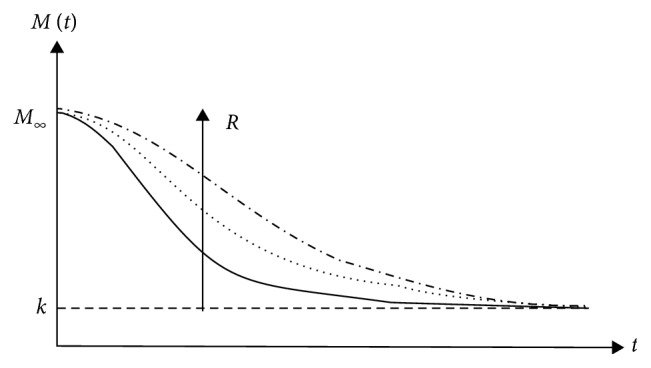
Release curves of the same *k*〈*M*_*∞*_ and various *R* described by the solution of the logistic type equation (1).

**Figure 5:**
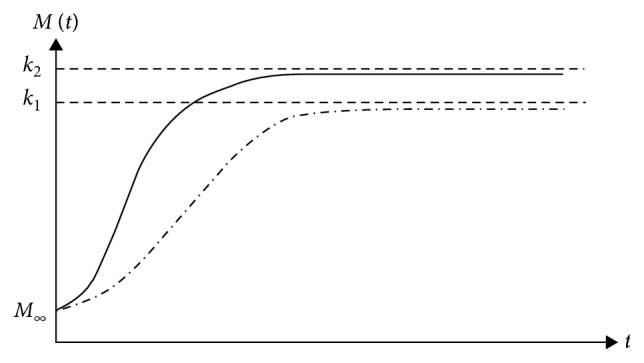
Release curves of the same *R* and various *k*, with *k*〉*M*_*∞*_, described by the solution of the logistic type equation (1).

**Figure 6:**
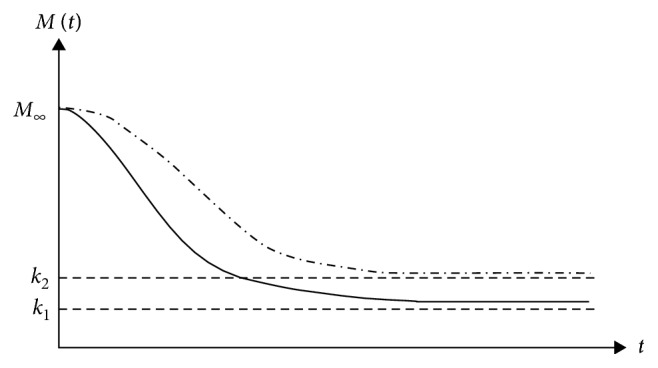
Release curves of the same *R* and various *k*, with *k*〈*M*_*∞*_, described by the solution of the logistic type equation (1).

The absence of archeology/history is specific to chaotic systems. Here, the behaviour of any such system is dictated by the relatively small number of nonlinear interactions between its structural units, and it has the fundamental property the “chaotic order.” Thus, the chaotic systems can be assimilated with a subset of complex systems without history. In such context, the following question arises: is it possible like a chaotic polymer-drug matrix, without history, to mimic the complex polymer-drug matrix, a system with history? The answer is affirmative only to the extent that the chaotic system (chaotic polymer-drug matrix) is “assigned” a type of archeology/history. In this regard, let us first notice that the polymer-drug matrix as a chaotic system can “support” a release mechanism based on the nondimensional logistic type law [19]:

(1) $$\begin{array}{c} \frac{dM}{dt}=RM\left(1-\frac{M}{K}\right), \end{array}$$

with

(2) $$\begin{array}{c} R>0,\;K>0,\;M\left(0\right)=M_{\infty }, \end{array}$$

where *M* is the amount of drug released at time *t*, *M*_*∞*_ is the amount of drug released after an infinitely long time that can be approximated with the amount of drug initially loaded into the polymer matrix, and *R* and *K* are the structure constants specific to the release mechanism. Indeed, these “situations” are verified directly taking into account that the solution of equation (2) in the following form:

(3) $$\begin{array}{c} M\left(t\right)=\frac{M_{\infty }K}{M_{\infty }+\exp\left(-Rt\right)\left(K-M_{\infty }\right)}, \end{array}$$

can describe, for different values of *R*, *K*, and *M*, complex release dynamics for various temporal sequences (see Figures 3–6).

A possible procedure for assigning archeology/history to polymer-drug matrix is through the multifractality of the release curves (continuous curves with varying degrees of nondifferentiability, hereinafter referred to as multifractal release curves). Consequently [20, 21], (i) any physical variable describing the release dynamics becomes dependent both on spatial and time coordinates and on the scale resolutions of multifractal type; mathematically, such a situation is possible considering that any physical variable acts as the limit of a family of functions, the functions being nondifferentiable for null scale resolutions of multifractal type and differentiable for non-null scale resolutions of multifractal type; (ii) the “laws” describing the release dynamics become invariant with respect to spatial and temporal coordinates transformations, as well as with transformations of scale resolutions of multifractal type; (iii) the constrained release dynamics on continuous and differentiable release curves in an Euclidean space are substituted with release dynamics, free of any constraints, on continuous release curves with different degrees of nondifferentiability (release curves of multifractal type) in a multifractal space; moreover, the release curves of multifractal type works also as geodesics of the multifractal space; (iv) between two points of a multifractal space, there are an infinity of geodesics and, thus, of multifractal release curves [20, 21].

In such a context, the indiscernibility of the release curves is a result of multifractalisation by multistocasticization—simultaneously, any monofractalization from the singularity spectrum of the release dynamics being compatible with a stochastic process. However, the discernibility of the release curves involves a selection process (obviously at scale resolution of finite multifractal type) of multifractal space geodesics based on a measurement process, from the multitude of multifractals, manifested at a given moment in the singularity spectrum of the release dynamics; only one confers globality through the type of the stochastic process.

In such a context was analyzed the dynamics of release from different types of drug delivery systems, such as microparticles [22], nanoparticles [23], and carriers for the transfer of genetic material [24], proving thus its universal character [25]. Its universal character has been enhanced by the application in the study of various chemical and physical systems, such as plasma [26–29] and nanofluids [30] but also various other dynamics [31–43].

In these conditions, the release dynamics of the polymer-drug matrix, considered now as complex system, can be described by the generalized nondimensional logistic type law:

(4) $$\begin{array}{c} \frac{dM}{dt}=RM\left[1-{\left(\frac{M}{K}\right)}^{F\left(\alpha \right)}\right], \end{array}$$

with

(5) $$\begin{array}{c} M=M\left[t,\mu ,F\left(\alpha \right)\right],\\ R=R\left[\mu ,F\left(\alpha \right)\right],\\ K=K\left[\mu ,F\left(\alpha \right)\right],\\ M\left[0,\mu ,F\left(\alpha \right)\right]=M_{\infty }\left[\mu ,F\left(\alpha \right)\right],\\ \alpha =\alpha \left(D_{F}\right), \end{array}$$

where *M* is the amount of released drug of multifractal type at time *t*, *M*_*∞*_ is the amount of drug released of multifractal type, after an infinite time, equal to the amount of drug initially loaded into the polymer matrix, *R* and *K* are the structure constants specific to the release mechanism of multifractal type, *μ* is the scale resolution of multifractal type, and *F*=*F*(*α*) is the singularity spectrum of singularity index *α*, an index functionally dependent of the fractal dimension *D*_*F*_ of the release curve. Let us note that the singularity spectrum *F*(*α*) will allow the identification of some “universality classes” in the field of “dynamical systems with release.”

In order to solve equation (4), let us substitute *V*[*t*, *μ*, *F*(*α*)]=1/*M*[*t*, *μ*, *F*(*α*)] with *V*[0, *μ*, *F*(*α*)]=*V*_0_[*μ*, *F*(*α*)]. We will have the following equation:

(6) $$\begin{array}{c} \frac{dV}{dt}=-\frac{1}{M^{2}}\frac{dM}{dt}=-\frac{RM}{M^{2}}\left[1-{\left(\frac{M}{K}\right)}^{F\left(\alpha \right)}\right], \end{array}$$

(7) $$\begin{array}{c} \frac{dV}{dt}=-\frac{R}{M}+\frac{RM^{F\left(\alpha \right)-1}}{K^{F\left(\alpha \right)}}=-RV+\frac{RV^{1-F\left(\alpha \right)}}{K^{F\left(\alpha \right)}}. \end{array}$$

Next, let us introduce a new substitution given by *Z*=*V*^*F*(*α*)^ with *Z*_0_=*V*_0_^*F*(*α*)^. Thus, a multifractal differential equation of Bernoulli type is obtained in the following form:

(8) $$\begin{array}{c} \frac{dZ}{dt}=F\left(\alpha \right)V^{F\left(\alpha \right)-1}\frac{dV}{dt}=F\left(\alpha \right)V^{F\left(\alpha \right)-1}\left[-RV+\frac{RV^{1-F\left(\alpha \right)}}{K^{F\left(\alpha \right)}}\right]=-F\left(\alpha \right)RV^{F\left(\alpha \right)}+F\left(\alpha \right)\frac{R}{K^{F\left(\alpha \right)}}=-F\left(\alpha \right)RZ+F\left(\alpha \right)\frac{R}{K^{F\left(\alpha \right)}}. \end{array}$$

This equation admits the following solution:

(9) $$\begin{array}{c} Z\left[t,F\left(\alpha \right)\right]=Z_{0}\exp\left[-F\left(\alpha \right)Rt\right]+\frac{1}{K^{F\left(\alpha \right)}}\left\{1-\exp\left[-F\left(\alpha \right)Rt\right]\right\}. \end{array}$$

The above solution has, in the variable *V*, the following expression:

(10) $$\begin{array}{c} V\left[t,\mu ,F\left(\alpha \right)\right]={\left[V_{0}^{F\left(\alpha \right)}\exp\left[-F\left(\alpha \right)Rt\right]+\frac{1}{K^{F\left(\alpha \right)}}\left\{1-\exp\left[-F\left(\alpha \right)Rt\right]\right\}\right]}^{1/F\left(\alpha \right)}. \end{array}$$

While in the variable *M*, it becomes

(11) $$\begin{array}{c} M\left[t,\mu ,F\left(\alpha \right)\right]={\left[\frac{1}{M_{\infty }^{F\left(\alpha \right)}}\exp\left[-F\left(\alpha \right)Rt\right]+\frac{1}{K^{F\left(\alpha \right)}}\left\{1-\exp\left[-F\left(\alpha \right)Rt\right]\right\}\right]}^{-\left(1/F\left(\alpha \right)\right)}=M_{\infty }K{\left\{M_{\infty }^{F\left(\alpha \right)}+\left[K^{F\left(\alpha \right)}-M_{\infty }^{F\left(\alpha \right)}\right]\exp\left[-F\left(\alpha \right)Rt\right]\right\}}^{-\left(1/F\left(\alpha \right)\right)}. \end{array}$$

The stationary points can be calculated as above. They are

(12) $$\begin{array}{c} \bar{M_{1}}=0,\\ \bar{M_{2}}=K. \end{array}$$

The derivative of function *F*[*M*, *R*, *K*, *F*(*α*)]=*RM*[1 − (*M*/*K*)^*F*(*α*)^] is

(13) $$\begin{array}{c} F^{\prime }\left(M\right)=R\left\{1-\left[1+F\left(\alpha \right)\right]{\left(\frac{M}{K}\right)}^{F\left(\alpha \right)}\right\}. \end{array}$$

As $F^{\prime }\left(\bar{M_{1}}\right)=R\rangle 0$, $\bar{M_{1}}=0$ is an unstable stationary point. In turn, the stationary point $\bar{M_{2}}=K$ is asymptotically stable because $F^{\prime }\left(\bar{M_{2}}\right)=-F\left(\alpha \right)R\langle 0$. Concluding, the realized mass will tend to the carrying capacity over time.

The inflexion point of the solution is given by means of condition:

(14) $$\begin{array}{c} F^{\prime }\left(0\right)=0, \end{array}$$

which implies the following expression:

(15) $$\begin{array}{c} M=\frac{K}{{\left[F\left(\alpha \right)+1\right]}^{1/F\left(\alpha \right)}}. \end{array}$$

## 4. Results

**Figure 7:**
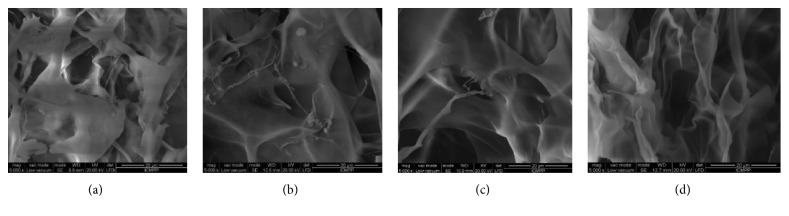
SEM microimages of the studied drug delivery systems: (a) A2D; (b) A3D; (c) A4D; (d) A5D.

Applying the procedure of in situ hydrogelation described in Section 2, a series of 4 drug delivery systems based on chitosan, nitrosalicylaldehyde, and diclofenac sodium salt, noted A2D–A5D (Figure 1), were prepared, in view of modelling their drug release characteristics. As the morphology of the drug delivery systems drastically influences the release mechanism, the A2D–A5D samples were firstly subjected to scanning electron microscopy measurements. As can be seen in Figure 7, the samples were porous, without visible DCF crystals, pointing for its submicrometric encapsulation. This has been attributed to the strong intermolecular forces which aroused between the polycationic chitosan and negatively charged DCF anion [9, 17, 44, 45].

**Figure 8:**
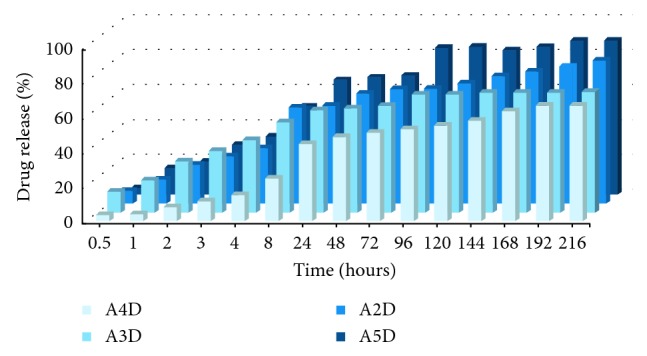
Monitoring of the DCF release from the A2D–A5D formulations.

The monitoring of the DCF release from the under study systems is represented in Figure 8. As can be seen, the release was significantly affected by the encapsulation pathway. From A2D and A5D systems, in which the DCF drug was confined as micrometric crystals, the release occurred faster, while from A3D and A4D systems, in which the drug was fine dispersed, the release produced slower. The encapsulation pathway played a key role in the total amount of the drug released from the matrix, too. As can be seen, after 8 hours, the A2D and A5D released almost entire amount of the drug, while the A3D and A4D only 70%. This can be explained by the stronger intermolecular forces developed in the case of the fine dispersion of the drug into the hydrogel matrix, forces which surpassed the interfacial ones developed in the case of dispersion of the drug as microcrystals [17].

The release data were fitted on the equations of five different standard (empirical/semiempirical) models [18]: *zero order*, *first order*, *Higuchi*, *Korsmeyer–Peppas*, and *Hixson–Crowell*.

**Figure 9:**
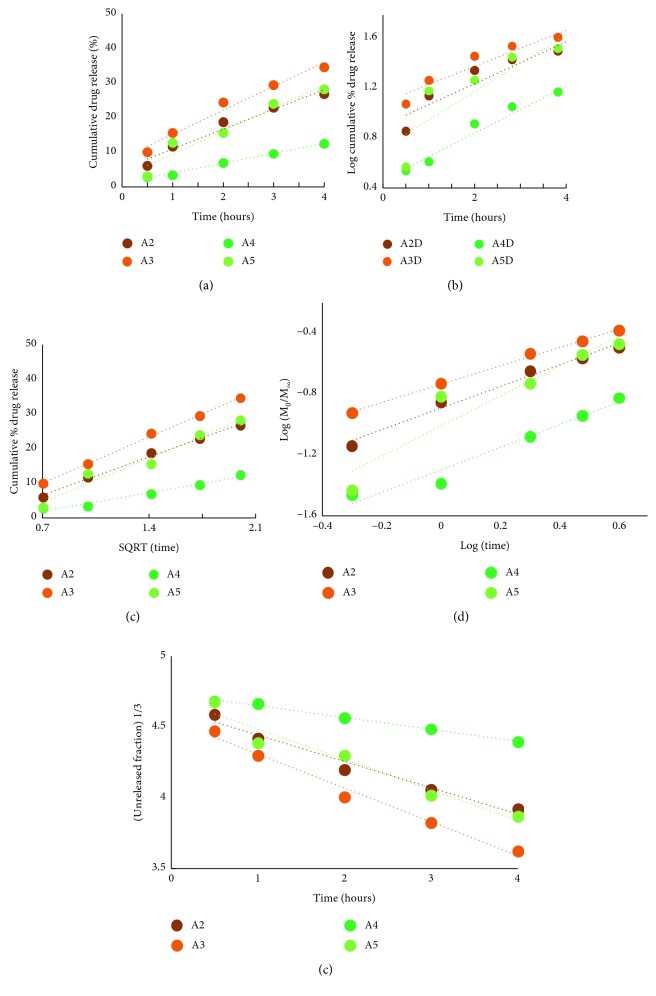
Linear forms of the zero order (a); first order (b); Higuchi (c); Korsmeyer–Peppas (d); and Hixson–Crowell (e) models applied for the release of DCF from the A2D-A5D formulations.

As can be seen in Figure 9, all the obtained release data proved a good fitting on the Hixson–Crowell model, indicating that the release mechanism is mainly controlled by the dissolution velocity and less by the diffusion through the matrix. On the other hand, in the case of the samples with a higher crosslinking density, a good fitting has been obtained also for the Higuchi and Korsmeyer–Peppas models, indicating that the drug release is also strongly influenced by the diffusion process through the hydrogel network.

**Figure 10:**
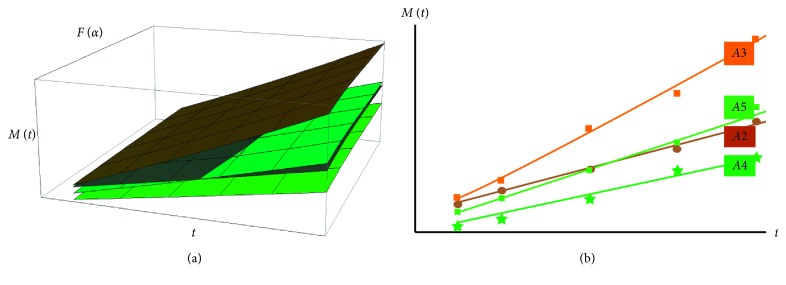
(a) 3D dependence of the amount of drug released, *M*, as a function of time *t*, and the global fractal dimension; (b) theoretical and experimental correlations.

Taking into account our theoretical model presented in Section 3, we present next, in Figure 10(a), the three dimensional qualitative dependence of the amount of drug released, *M*, as a function of time *t*, and the global fractal dimension, dictated through the singularity spectrum *F*(*α*). In such conjecture, a time linear dependence is observed. By a convenient choice of the normalization parameters, the correlations with the experimental data are illustrated in Figure 10(b).

## 5. Discussion

In the present paper, release dynamics are analyzed, both theoretical and experimental, for a special polymer-drug matrix, based on standard models (empirical/semiempirical), zero order, first order, Higuchi, Korsmeyer–Peppas, and Hixson–Crowell, and on the basis of a new model given by a logistic type law.

Our approach is based on the following: (i) first, the logistic type law (1) is proposed in the description of the release dynamics. In this relation, the term *RM* specifies the difference between the rate of occurrence and the rate of disappearance of the released drug mass, while the term *RM*^2^/*K* limits the increase in drug mass released by typical processes such as the degradation of the polymeric matrix. Such a release system is a chaotic one: its behaviour is imposed by a relatively small number of nonlinear interactions between its structural units, it does not have archeology/history (or if it contains a “intrinsic” one, it is freezed—it does not manifest itself), and it has as the fundamental property the “chaotic order”; (ii) then, a generalization of logistic type law (1) is proposed in the form of relation (4) in order to describe the release dynamics, assuming that the release curves are of the multifractal type. Such a choice has several obvious implications: (a) the drug mass released given by (4) becomes dependent both on time coordinate and on the scale resolutions of multifractal type. Mathematically, such a situation is possible considering that the released drug mass, given by (4), acts as the limit of a family of functions, the functions being nondifferentiable for null scale resolutions of multifractal type and differentiable for non-null scale resolutions of multifractal type; (b) the release dynamics described by relation (4) is invariant both in relation to temporal transformations and in relation to scale resolution transformations; (c) the constrained release dynamics on multifractal type curves in an Euclidian space is substituted with release dynamics, free of any constraints in a multifractal space, etc.

## 6. Conclusion

A mathematical model that intends to describe the dynamics of controlled drug release systems should allow (a) to predict the drug release kinetics and the phenomena involved, avoiding thus repetitive time and money expensive experiments; (b) to optimize the drug release kinetics; (c) to estimate the effect of the polymer matrix design parameters, i.e., shape, size, and composition on the drug release kinetics; (d) to predict the global therapeutic efficiency and drug safety; and (e) overall, to design a new drug delivery system depending on the release kinetics imposed by the therapeutic requirements.

So far, successful mathematical models designed to describe the dynamics of the drug controlled release system have been developed based on either an almost intuitive selection of dominant phenomena relative to the polymer matrix configuration and geometry or just by analysing the experimental results, resulting in a wide variety of empirical/semiempirical models such as the diffusion model, the zero-kinetic model, the Higuchi model, the Korsemeyer–Peppas model, the Hixon–Crowell model, the Weibull model, the Baker Lonsdale model, the Hopfenberg model, the Gompertz model, the sequential layer model, the Peppas–Sahlin model, etc.

In our opinion, this diversity of models demonstrates the inability of differential and integral mathematical procedures to describe the complexity of controlled drug release dynamics. Thus, the success of the abovementioned models should be understood only sequentially/progressively in domains where differentiability and integrity are still valid. However, the differential and integral mathematical procedures “suffer” when one wants to describe the dynamics of drug controlled release systems, dynamics involving both nonlinearity and chaos.

The model developed in this paper is precisely intended to substitute the standard mathematical procedures, i.e., of differential and integral types, with nondifferential and nonintegrative mathematical procedures, applied in describing the dynamics of controlled drug release systems. In such a conjecture, we first developed the logistic model of dynamics of drug controlled release systems (where the system is only chaotic, without memory, behaviour dictated by a relatively small number of nonlinear interactions between its entities and still subject to differential and integral mathematical procedures). Then, by extension, we developed the multifractal logistic model for the dynamics of controlled drug release systems (where the system is complex, with memory, its behaviour being imposed by a large number of nonlinear interactions between its entities, subjected only to nondifferential and nonintegrative mathematical procedures); such a description proves to be reducible to the dynamics of drug controlled release systems at various scale resolutions.

The model was finally validated on the basis of the experimental results on diclofenac sodium salt release from nitrosalicyl-imine-chitosan hydrogels, confirming thus that complex systems, i.e., polymer-drug matrices, are pseudo-intelligent systems, with memory. In such an approach, phenomena as drug diffusion, swelling, and degradation of the polymeric matrix can be described as dynamics of pseudo-intelligent materials at various scale resolutions.

## Data Availability

Previously reported experimental data were used to support this study and are available at https://doi.org/10.1016/j.jcis.2018.10.048.
